# The Efficacy and Safety of Roxadustat for Anemia in Patients With Chronic Kidney Disease: A Meta-Analysis

**DOI:** 10.3389/fphar.2022.779694

**Published:** 2022-04-26

**Authors:** Lijun Wang, Heng Yin, Liling Yang, Fenglian Zhang, Song Wang, Dan Liao

**Affiliations:** Mianyang Central Hospital, School of Medicine, University of Electronic Science and Technology of China, Mianyang, China

**Keywords:** Anemia, chronic kidney disease, meta-analysis, roxadustat, CKD, FG-4592

## Abstract

**Background:** Chronic kidney disease (CKD) is a global public health problem, and anemia is a common complication in CKD patients. Roxadustat (FG-4592) is an oral hypoxia-inducible factor (HIF) stabilizer. Roxadustat has been shown in studies to keep up with and increase hemoglobin better than placebo or erythropoietin. The purpose of this meta-analysis was to assess the efficacy and safety of roxadustat.

**Methods:** We searched CBM, CNKI, VIP, Wanfang Database, PubMed, Cochrane Library, Embase, and Web of Science for randomized controlled trials of roxadustat for the treatment of anemia in CKD patients. The papers were screened using rigorous criteria and their quality was assessed using the Cochrane 5.1.0 assessment manual for randomized controlled trials (RCTs). RevMan 5.3 was used to extract and synthesize data for meta-analysis.

**Results:** There were 8 RCTs (7 articles) in all, and 1,364 patients with chronic kidney disease anemia were involved. The overall quality of the studies included was satisfactory. The meta-analysis findings revealed that roxadustat can considerably enhance hemoglobin, transferrin, and total iron binding capacity (TIBC) in both dialysis-dependent (DD) and non-dialysis-dependent (NDD) patients: Hemoglobin (Hb): DD: [SMD = 0.23, 95% CI (0.01, 0.44), *p* = 0.04], NDD: [SMD = 2.08, 95% CI (1.23, 2.93) *p* < 0.000001]; transferrin: DD: [SMD = 0.78, 95% CI (0.24, 1.32), *p* = 0.004], NDD: [SMD = 1.37, 95% CI (0.76, 1.98), *p* < 0.0001]; TIBC: DD [SMD = 0.97, 95% CI (0.64, 1.29), *p* < 0.00001], NDD [SMD = 1.34, 95% CI (0.9, 1.78), *p* < 0.00001]. After roxadustat therapy, patients’ serum iron levels were considerably higher in the dialysis group than in the control group, but there was no significant change in the NDD group [SMD = 0.42, 95% CI (0.27, 0.57), *p* < 0.00001]. In the NDD group, hepcidin, ferritin, and transferrin saturation (TSAT) were significantly reduced after roxadustat treatment: Hepcidin [SMD = −1.59, 95% CI (−2.69, −0.49), *p* = 0.005], ferritin [SMD = −0.51, 95% CI (−0.72, 0.3) *p* < 0.00001], TSAT [SMD = −0.41, 95% CI (−0.62, 0.2), *p* < 0.0001]. In terms of safety, adverse events (AE) [SMD = 1.08, 95% CI (0.98, 1.18) *p* = 0.11] and serious adverse events (SAE) [SMD = 1.32, 95% CI (0.97, 1.9) *p* = 0.08] were not significantly different between the two groups.

**Conclusion:** Roxadustat can improve anemia in NDD patients with chronic kidney disease, and its short-term safety was comparable to that of the comparison group.

## 1 Introduction

Renal anemia is a common complication in patients with CKD. Renal anemia has long been thought to be an independent risk factor that affects CKD patients’ prognoses and increases their risk of cardiovascular complications and death (Portolés et al., 2013; Sato et al., 2017). The incidence of anemia in the young and middle-aged nondialysis population with chronic kidney disease stages 3–5 is 28%, while the incidence of anemia in the young and middle-aged dialysis population with chronic kidney disease stage 5 is 53.9% (St Peter et al., 2018). The incidence of anemia in the elderly nondialysis population with chronic kidney disease stage 5 is as high as 72.8%; among those getting dialysis, the incidence is significantly greater. In China, the prevalence of renal anemia is significant, but awareness, treatment, and compliance rates are poor. Failure to achieve standard Hb levels in both dialysis and nondialysis CKD patients will accelerate the progression of CKD. Current phase I, II, and III clinical trial findings for the new oral medication, hypoxia-inducible factor prolyl hydroxylase inhibitor (HIFPHI) roxadustat, suggest that the medicine can cure renal anemia (Dhillon, 2019). Despite the fact that various clinical trials and meta-analyses on the clinical effectiveness of roxadustat have been conducted in China and elsewhere, the results of the meta-analyses were inconsistent. The publications thoroughly examined the efficacy and safety of roxadustat in the treatment of anemia, whereas Liu et al. (2021) focused mostly on its safety. We conducted a systematic analysis of published randomized controlled trials to assess the safety and efficacy of roxadustat for the treatment of renal anemia in patients with chronic kidney disease, as well as to offer evidence-based medical evidence for clinical management.

## 2 Materials and Methods

### 2.1 Search Strategy

From conception to May 2021, we searched PubMed, Embase, Cochrane Library, Web of Science, SinoMed, China National Knowledge Infrastructure, WanFang, and VIP Information databases for clinical studies examining roxadustat for anemia in CKD patients. The search phrases were “roxadustat,” “FG-4592,” “anemia,” “chronic kidney disease,” “CKD,” and “kidney disease.” We also searched ClinicalTrials.gov and the references in selected papers and reviews for additional relevant material.

### 2.2 Inclusion Criteria

1) The individuals with chronic kidney disease anemia who were included in the study met the following criteria: ≥18 years old; CKD 3-5, in accordance with World Health Organization (WHO) anemic diagnostic criteria (KDIGO Clinical Practice Guideline Working Group, 2012). Accept or refuse dialysis; Receiving maintenance hemodialysis three times a week for 12 weeks or longer; Before joining the trial, the HIF-PHIs preparation roxadustat was not used; gender, race, and area are not restricted. Randomized controlled studies, both published and unpublished.
2) Intervention measures: ① Experimental group: Roxadustat; ② Control group: 1. Placebo; 2. Erythropoietin (EPO) or Epoetin alfa; 3.Darbepoetin alfa (DA).
3) Outcome indicators: main indicators: the change of the average (Hb, Iron, transferrin, hepcidin, ferritin, TSAT)levels from baseline to the end, serious adverse events, adverse events.

### 2.3 Exclusion Criteria

1) Duplicate literature and trials for clinical registration; 2) Review category and letter literature; 3) Systematic reviews and Meta analysis that are not related to the research topic; 4) Irrelevant literature (including animal trials and non-chronic subjects) The literature and clinical registration studies and intervention measures of renal anemia are Placebo or epoetin alfa literatures); 5) Conference abstracts and full texts are not available; 6) Non-random studies: including individual cases, case series studies, and cross-sections studies and non-randomized clinical registration trials; 7) The clinical trials and literature that the objects are not roxadustat; 8) No data clinical registration trials.

### 2.4 Data Extraction

Using a standardized form, two reviewers (Lijun Wang and Heng Yin) independently retrieved data from original trial reports. Data extracted included study characteristics (first author, publication year, single or multicenter, sample size, intervention and control, treatment period and duration of follow-up), patient characteristics (inclusion criteria, background treatments, mean age, proportion of men, baseline weight, and baseline Hb levels), reported outcomes (Hb, transferrin, hepcidin, ferritin, TSAT, AEs and SAEs), and methodology information.

### 2.5 Quality Assessment

Two reviewers independently reviewed the literature using the inclusion and exclusion criteria, and the included papers were appraised using the RCT quality evaluation criteria. When a disagreement arises throughout the screening and assessment process, it will be discussed, mediated, and resolved with the help of a third party.

The Cochrane Collaboration’s tool was used to assess the risk of bias in RCTs. The assessments were conducted separately by two investigators; disagreements were reviewed with a third party and resolved by consensus. Furthermore, the Grading of Recommendations Assessment, Development, and Evaluation (GRADE) framework was used to assess the quality of evidence contributing to each estimate, which characterizes the quality of a body of evidence for the primary outcomes based on study limitations, imprecision, inconsistency, indirectness, and publication bias.

### 2.6 Literature Selection and Quality Evaluation

Two reviewers independently reviewed the literature using the inclusion and exclusion criteria, and the included papers were appraised using the RCT quality evaluation criteria. When a disagreement arises throughout the screening and assessment process, it will be discussed, mediated, and resolved with the help of a third party.

### 2.7 Methodological Quality Evaluation of Included Studies

The quality evaluation tool recommended by Cochrane Handbook 5.1.0 (Cochrane, 2011)was used to evaluate the included studies, including seven items: 1) Whether the random sequence generation method is appropriate; 2) The hiding method of the allocation sequence: whether it is sequential or Containers with the same code or sealed in opaque envelopes, etc.; 3) Blind the researcher and subjects and ensure that the blinding is not destroyed during the trial; 4) Blind evaluation of the research outcome; 5) Result data Completeness: whether missing data affects the outcome; 6) selective reporting of research results; 7) other biases: whether there are conflicts of interest, fraud and deceptive behavior. Each document makes judgments of “yes” (low risk), “no” (high risk), and “unclear” (unclear) for the above seven items.

### 2.8 Statistical Analysis

The chi-square test was employed to examine the study heterogeneity. Use *I* ^*2*^ and *P* to undertake a quantitative study of the collected literature’s statistical heterogeneity. If *I* ^*2*^ < 50%, it means that there is statistical homogeneity among the studies, and the fixed-effects model is used in the result analysis; if *I* ^*2*^ ≥ 50%, it means that there is statistical heterogeneity among the studies, and the source of the heterogeneity will be further investigated, and subgroup analysis will be performed based on the factors that may cause the heterogeneity (Higgins and Thompson, 2002). The random effects model is employed for analysis in studies that still cannot remove statistical heterogeneity and have no clear clinical heterogeneity, and the difference is statistically significant with *p* < 0.05.

Use RevMan5.3 program to do Meta analysis on all RCT data that satisfy the requirement, choose relative risk (Risk Ratio, RR) and its 95% confidence interval (Confidence Interval, CI) to represent binary variables, and choose standard mean. Continuous variables are described using the Standardized Mean Difference (SMD) and its 95% confidence interval (CI). Sensitivity analysis assesses heterogeneity changes using an alternative analysis of the random effects model and the fixed effects model, and it summarizes the results’ stability.

### 2.9 Publication Bias

Stata 12.0 software was used to perform Beeg’s and Egeer’s tests, with *p* < 0.05 indicating statistically significant differences in the data and likely publication bias.

## 3 Result

1) Excluded by reading the title and abstract: 121 reviews, 26 repeated studies, 18 animal experiments, 27 non-chronic kidney disease anemic literature, 4 case studies, 43 clinical trials, and literature in which the objectives are not mentioned. 40 conference papers, Roxadustat there are no 19 publications on pharmacokinetics. 2) Excluded from consideration by reading the complete text: There were eight non-random studies (case reports, case series reports, cross-sectional studies), and eventually 9 RCTs.

**FIGURE 1:**
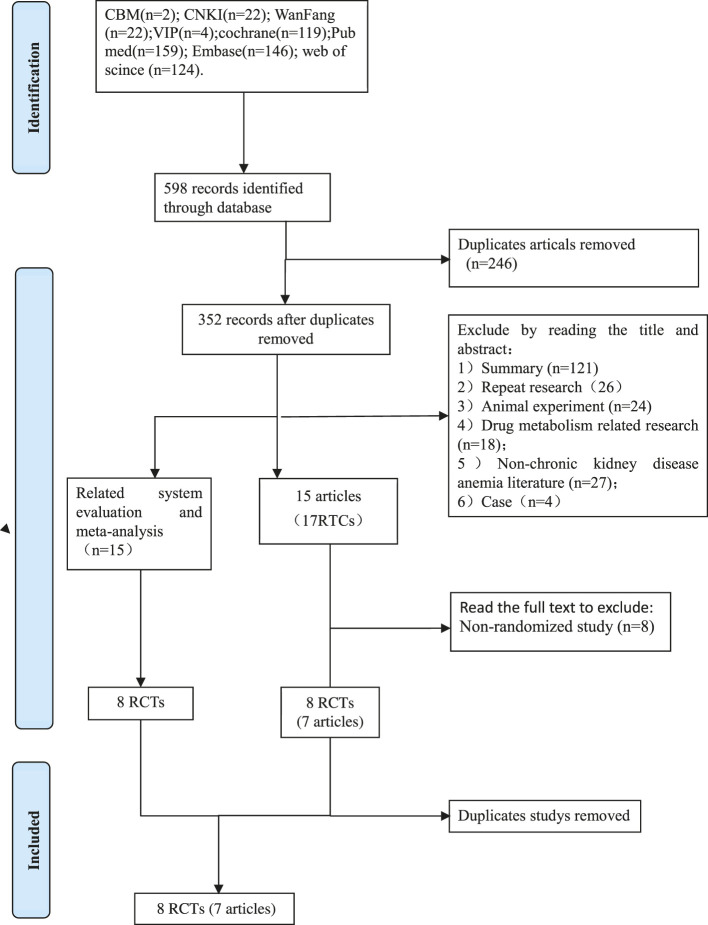
The PRISMA flow chart for the included studies.

Process for retrieving certain documents (Figure 1).

### 3.1 Characteristic Table of Included Literature

From 2015 through 2020, nine randomized controlled trials (RCTs) were included. The study comprised 1,364 chronic kidney disease anemia patients from the United States, Japan, and China. With 918 patients, the experimental group received roxadustat, whereas the control group received either a blank control or recombinant human erythropoiesis. There are 396 vegetarian patients ranging in age from 18 to 80 years. The typical roxadustat dosages are (0.7–2.25 mg/kg)/po tiw and 50–120 mg/po tiw. The purpose of this study is to examine the efficacy and safety of roxadustat in the treatment of patients with chronic renal disease, with a follow-up period ranging from 8 to 24 W. To investigate the utilization of roxadustat in the treatment of anemia in chronic kidney disease, the experimental and control groups were compared using markers such as hemoglobin, transferrin, hepcidin, ferritin, TSAT, TIBC, serum iron, and the occurrence of adverse events. The following studies were found to be effective and safe: (Table 1).

**TABLE 1:** Information of the included studies.

Study	Country	Object		Intervention			Follow up	Outcome
		E/C	Age (year)	E	E (D/U/T)	C		
Besarab et al. (2015a)	United States	88/29	18–80	Roxadustat	0.7, 1.0, 1.5 mg/kg, 2.0 mg/kg/po tiw/4 W	Placebo	16 W	O3, O4, O5, O6, O7, O8, O9
Provenzano et al. (2016)	United States	108/36	18–75	Roxadustat	1.0, 1.5, 1.8 mg/kg, 2.0 mg/kg/po tiw/6–19 W	Epoetin alfa	8 W	O3, O4, O5, O6, O7, O8, O9
Chen et al. (2017)	China	91/30	18–80	—	(1.1–1.75 mg/kg1.5–2.25 mg/kg)/po tiw/8w	Placebo	8 W	O1, O2, O3, O4, O5, O6, O7, O8, O9
Chen et al. (2017)	China	96/22	18–80	Roxadustat	(1.1–1.8 mg/kg, 1.5–2.3 mg/kg)/po tiw/8w	Epoetin alfa	24w	O1, O2, O3, O4, O5, O6, O7, O8, O9
Akizawa et al. (2019)	Japan	80/27	20–74	Roxadustat	50, 70, 100 mg/po tiw/24 W	Placebo	12 W	O1, O2, O3, O4, O5, O6, O7, O8, O9
Chen et al. (2019a)	China	204/100	18–75	Roxadustat	100, 120 mg/po tiw/26 W	Epoetin alfa	24 W	O1, O2, O3, O4, O5, O6, O7, O8, O9
Chen et al. (2019b)	China	101/51	18–75	Roxadustat	70, 100 mg/po tiw/18 W	Placebo	24 W	O1, O2, O3, O4, O5, O6, O7, O8, O9
Akizawa et al. (2020a)	Japan	150/151	≥20	Roxadustat	70, 100 mg/po tiw/24 W	DA	12 W	O2, O3, O4, O5, O6, O7, O8, O9

E/C, Experimental group/Control group; D/U/T, Dosage/Usage/Treatment; O1, △Hb; O2, △transferrin; O3, △hepcidin; O4, △ferritin; O5, △TSAT; O6, △TIBC; O7, △Iron; O8, AEs; O9, SAEs.

### 3.2 Literature Quality Evaluation

**FIGURE 2:**
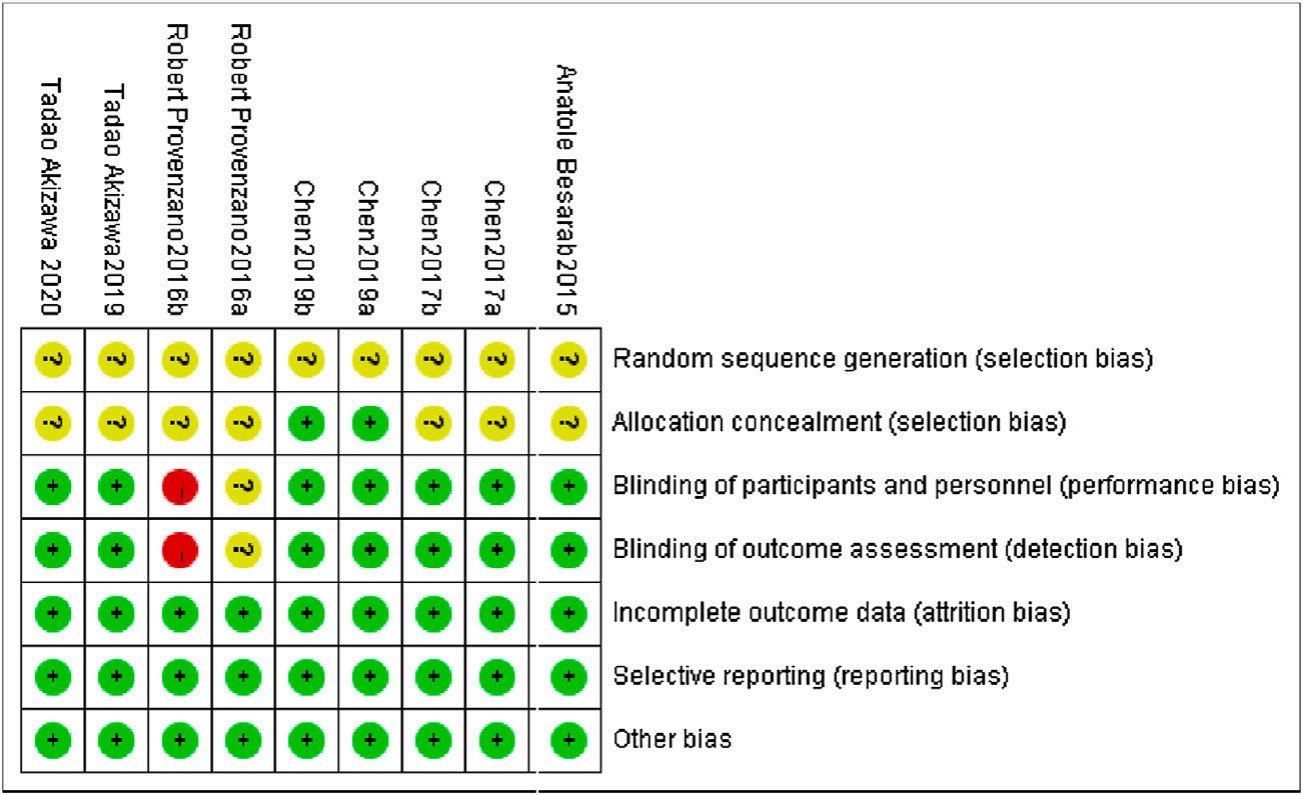
Risk of bias summary for included studies.

All of the studies included are clinical randomized controlled trials. The overall quality of the literature included in this analysis is high, and the included studies were appraised strictly in line with the Cochrane Handbook 5.1.0 quality evaluation standard. (Figure 2). The precise method of employing random sequence generation was not specifically mentioned in any of the RCTs.

The central distribution control system was employed for allocation concealment in both Chen et al. (2019a) and Chen et al. (2019b), while the remaining seven studies did not describe the allocation concealment technique. The use of blinding is not expressly described in Provenzano et al. (2016). Provenzano et al. (2016) is a single-blind experiment. All of the study’s outcome indicators had high data integrity, no selective reporting, and no additional biases.

## 4 Meta Analysis Results

### 4.1 Meta-Analysis

#### 4.1.1 Changes in Hb Levels From Baseline (Hb)

**FIGURE 3:**
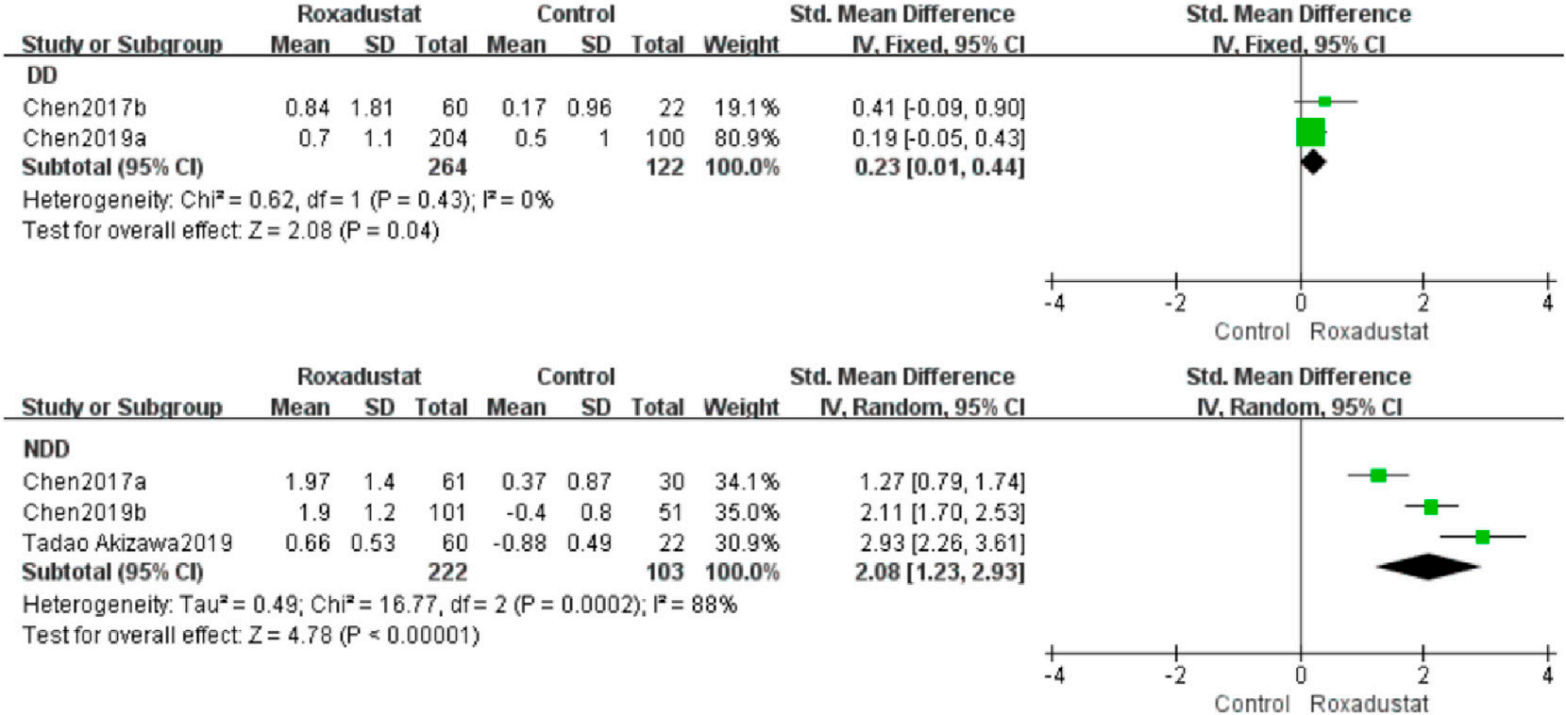
Changes in Hb levels from baseline (Hb).

A total of two investigations with 386 dialysis patients compared hemoglobin changes in the roxadustat group (*n* = 264) and the control group (*n* = 122) following therapy, and the overall effect size [SMD = 0.23, 95%CI (0.01, 0.44), *p* = 0.04] was statistically significant. The meta-analysis found that hemoglobin increased much higher in patients on regular dialysis who were given roxadustat than in the control group (Figure 3).

Three trials with 325 nondialysis patients evaluated hemoglobin changes in the roxadustat group (*n* = 222) and the control group (*n* = 103) after therapy and established the overall effect size [SMD = 2.08, 95% CI (1.23, 2.93), *p* < 0.000001], and the difference was statistically significant. The meta-analysis found that hemoglobin rose considerably higher in nondialysis patients treated with roxadustat than in the control group (Figure 3).

#### 4.1.2 Changes in Transferrin Levels From Baseline (Transferrin)

**FIGURE 4:**
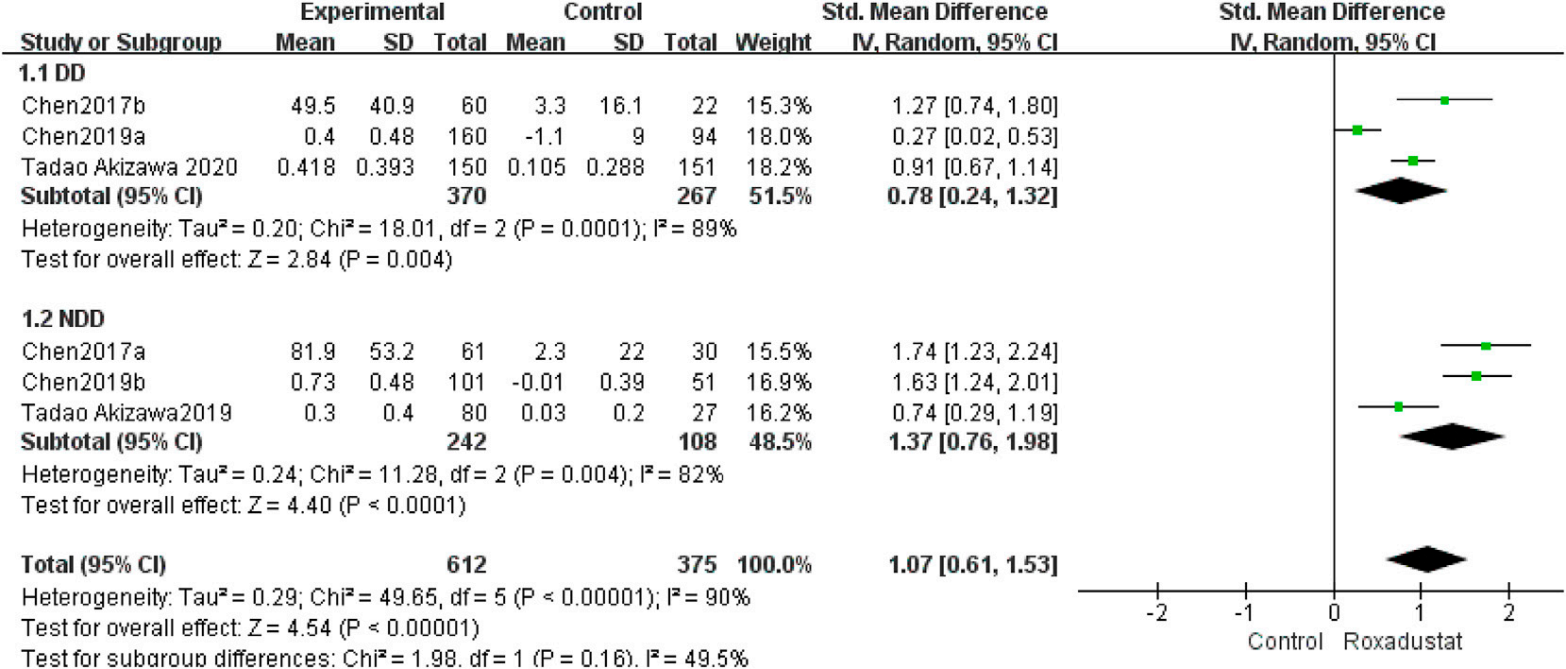
Changes in transferrin levels from baseline (transferrin).

A total of three studies with 637 dialysis patients evaluated transferrin variations between the roxadustat group (*n* = 370) and the control group (*n* = 267) and established the combined effect size [SMD = 0.78, 95% CI (0.24, 1.32), *p* = 0.004], and the difference was statistically significant. The meta-analysis found that, as compared to the control group, dialysis patients who took roxadustat had a substantial rise in transferrin (Figure 4).

Three studies with 325 nondialysis patients evaluated transferrin changes between the roxadustat group (*n* = 242) and the control group (*n* = 108) and found the overall effect size [SMD = 1.37, 95% CI (0.76, 1.98), *p* < 0.0001]; the difference was statistically significant. The meta-analysis found that, when compared to the control group, nondialysis patients treated with roxadustat had a substantial rise in transferrin (Figure 4).

#### 4.1.3 Changes in Hepcidin Levels From Baseline (Hepcidin)

**FIGURE 5:**
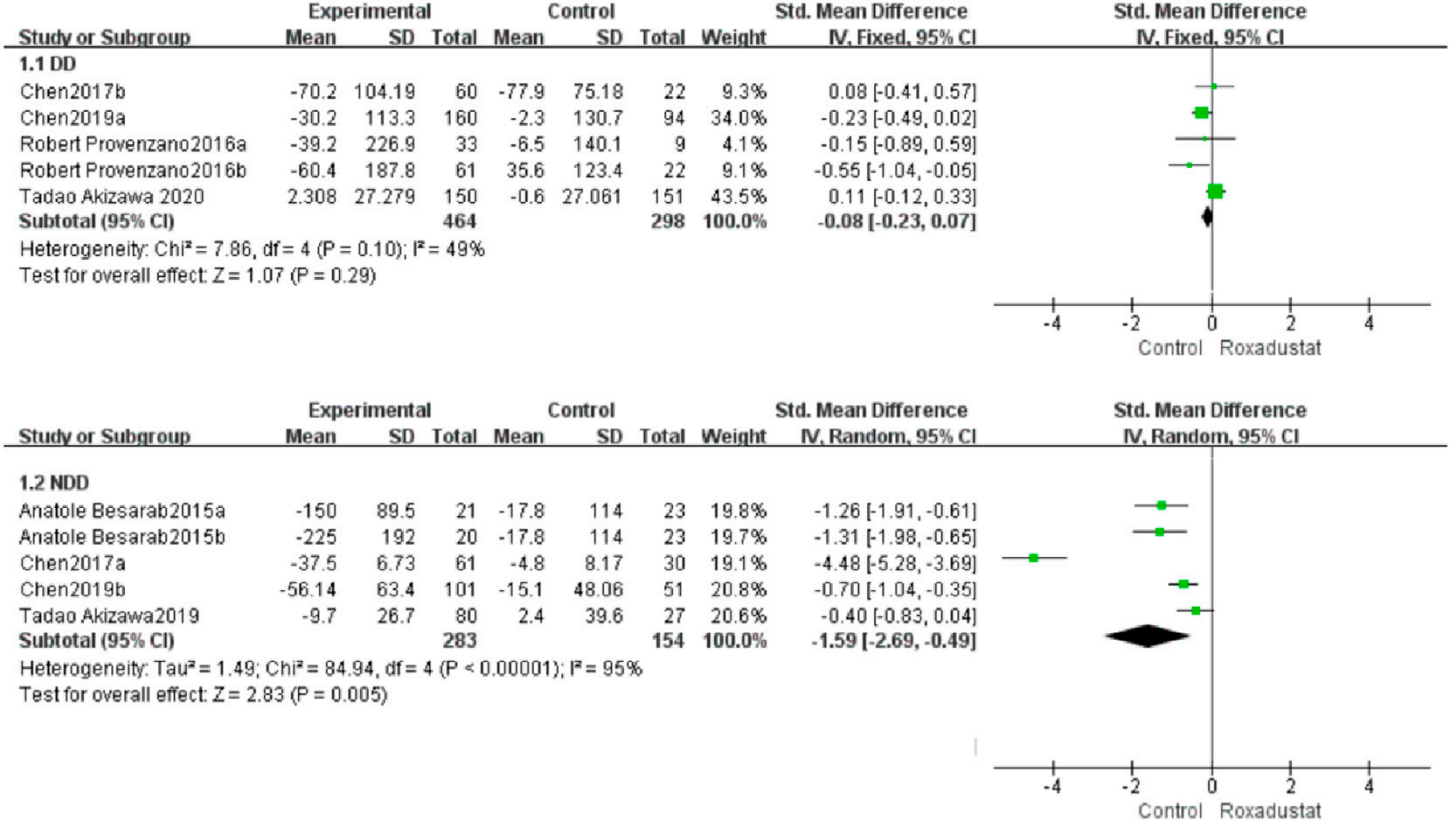
Changes in hepcidin levels from baseline (hepcidin).

A total of five trials with 762 dialysis patients compared hepcidin changes in the roxadustat group (*n* = 464) and the control group (*n* = 298) and calculated the overall effect size [SMD = −0.08, 95% CI (−0.23, 0.07), *p* = 0.29]. There was no statistically significant difference. The meta-analysis found no difference in hepcidin levels in DD patients following roxadustat therapy compared to the control group (Figure 5).

Five trials with 437 nondialysis patients evaluated hepcidin alterations in the roxadustat group (*n* = 283) and the control group (*n* = 154) and calculated the cumulative effect size [SMD = −1.69, 95% CI (−2.69, −0.49), *p* = 0.005]. There was a statistically significant difference. The meta-analysis found that following roxadustat therapy, hepcidin levels increased considerably more in NDD patients than in the control group (Figure 5).

#### 4.1.4 Changes in Ferritin Levels From Baseline (Ferritin)

**FIGURE 6:**
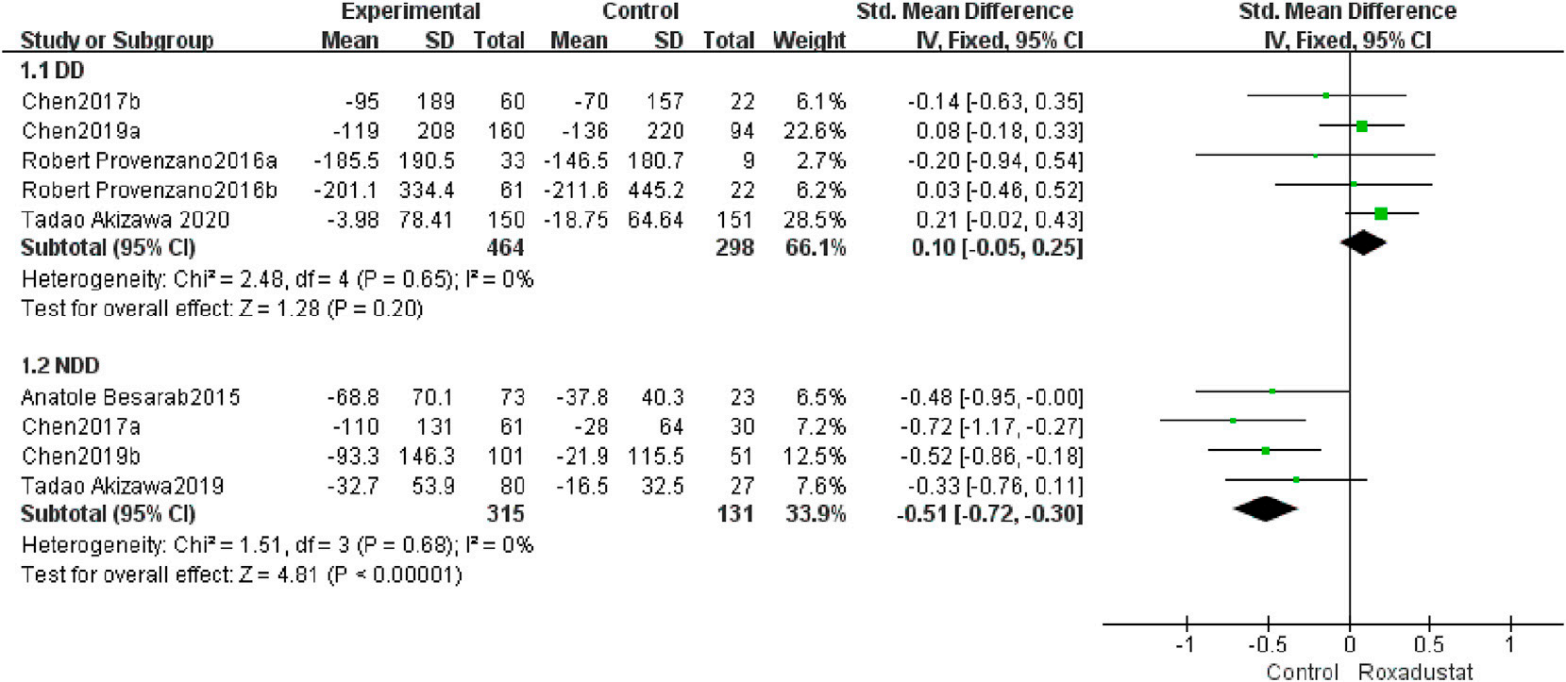
Changes in ferritin levels from baseline (ferritin).

A total of five trials with 762 dialysis patients examined ferritin variations in the roxadustat group (*n* = 464) and the control group (*n* = 298) following therapy, and the overall effect size [SMD = 0.10, 95% CI (−0.05, 0.25), *p* = 0.20] was not statistically significant. The meta-analysis found that ferritin levels in DD patients did not differ from those in the control group following roxadustat therapy (Figure 6).

A total of four trials with 446 non-dialysis patients compared the changes in ferritin in the roxadustat group (*n* = 315) and the control group (*n* = 131) following therapy, and the overall effect size [SMD = −0.51, 95% CI (−0.72, −0.3), *p* < 0.00001] is statistically significant. The meta-analysis found that ferritin levels increased considerably higher in NDD patients than in the control group following roxadustat medication (Figure 6).

#### 4.1.5 Changes in TAST Levels From Baseline

**FIGURE 7:**
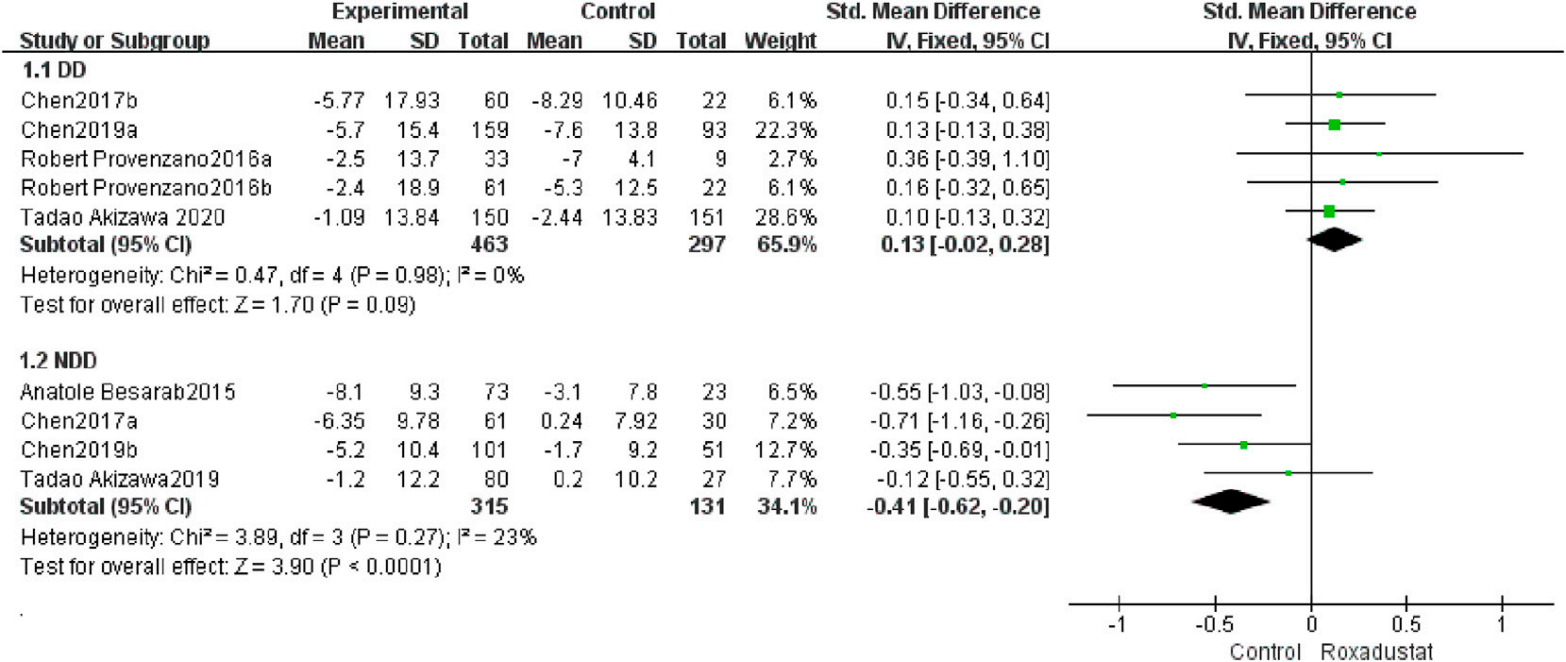
Changes in TSAT levels from baseline (TSAT).

A total of five trials with 762 dialysis patients examined the changes in TSAT following therapy in the roxadustat group (*n* = 464) and the control group (*n* = 297), and the cumulative effect size [SMD = 0.13, 95% CI (−0.02, 0.28), *p* = 0.09] was not statistically significant. According to the findings of the meta-analysis, there was no difference in TSAT in DD patients treated with roxadustat compared to the control group (Figure 7).

A total of four studies with 437 non-dialysis patients comparing changes in TSAT in the roxadustat group (*n* = 315) and the control group (*n* = 131) following therapy, and the overall effect size [SMD = −0.41, 95%CI (−0.62.69, −0.20), *p* < 0.0001] is statistically significant. The meta-analysis found that, as compared to the control group, TSAT increased considerably greater in NDD patients following roxadustat therapy (Figure 7).

#### 4.1.6 Changes in TIBC Levels From Baseline

**FIGURE 8:**
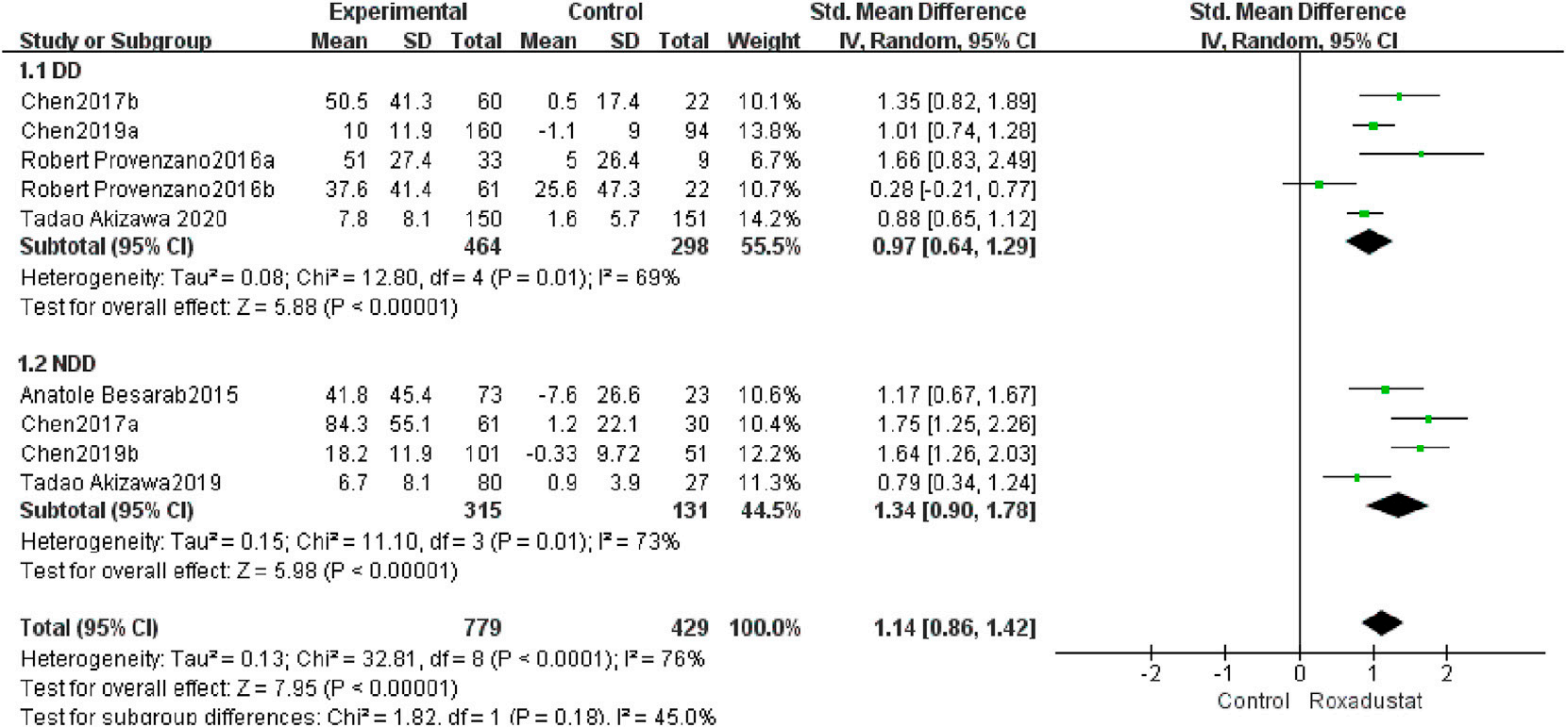
Changes in TIBC levels from baseline (TIBC).

A total of five trials with 762 dialysis patients compared the changes in TIBC following therapy in the roxadustat group (*n* = 464) and the control group (*n* = 298), and the cumulative effect size [SMD = 0.97, 95% CI (0.64, 1.29), *p* < 0.00001] was statistically significant. The meta-analysis found that TIBC rose considerably greater in DD patients following roxadustat therapy (Figure 8).

A total of four studies with 446 non-dialysis patients compared the changes in TIBC following therapy in the roxadustat group (*n* = 315) and the control group (*n* = 131), and the cumulative effect size [SMD = 1.34, 95 %CI (0.9, 1.78), *p* < 0.00001] was statistically significant. The meta-analysis found that TIBC rose considerably greater in NDD patients following roxadustat therapy (Figure 8).

#### 4.1.7 Changes in Iron Levels From Baseline

**FIGURE 9:**
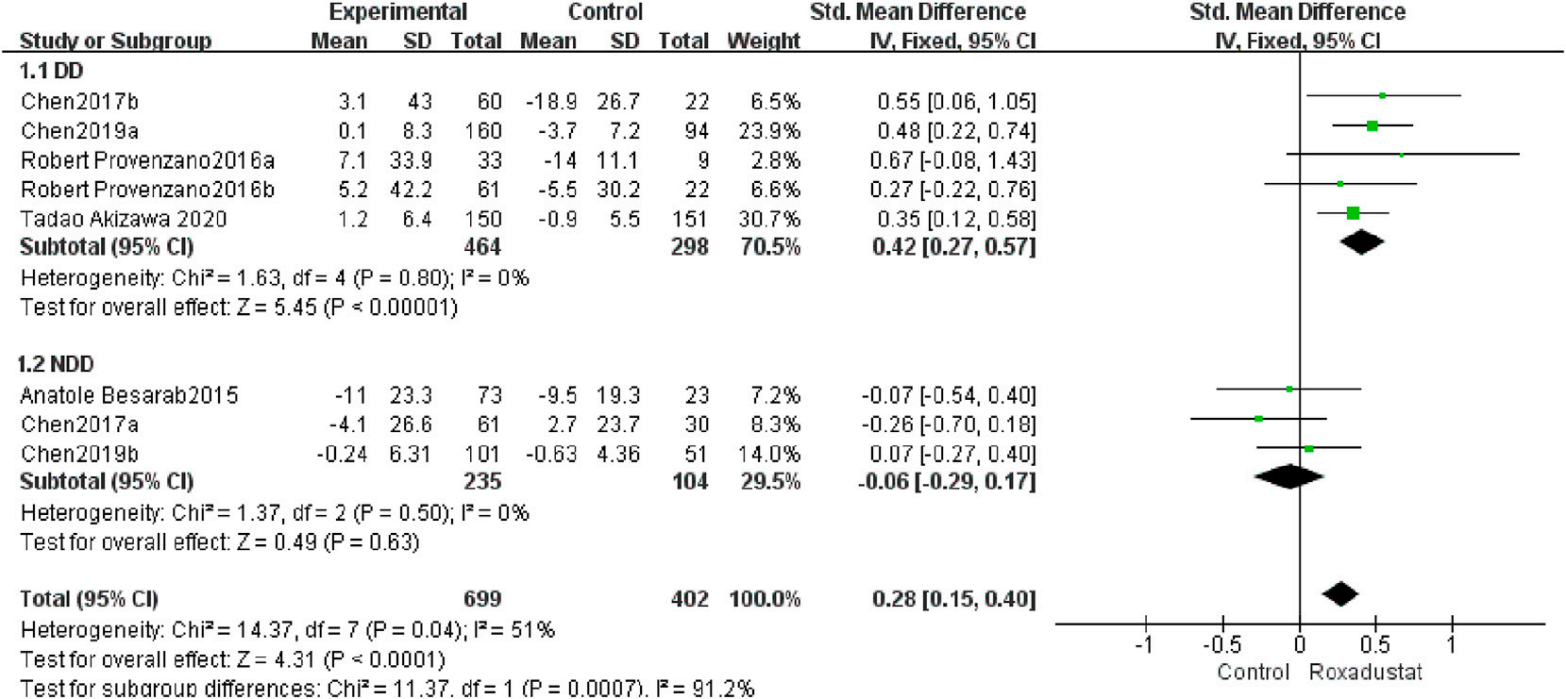
Changes in iron levels from baseline (iron).

A total of five studies with 762 patients examined the changes in iron following therapy in the roxadustat group (*n* = 464) and the control group (*n* = 298), and the cumulative effect size was not statistically significant [SMD = 0.42, 95% CI (0.27, 0.57), *p* < 0.00001]. According to the meta-analysis results, there was no change in serum iron in DD patients following roxadustat therapy (Figure 9).

A total of three trials with 339 patients comparing serum iron variations in the roxadustat group (*n* = 235) and the control group (*n* = 104) following therapy, and the overall effect size [SMD = −0.06, 95% CI (−0.29, 0.17), *p* = 0.63] is statistically significant. According to the findings of the meta-analysis, serum iron levels in NDD patients increased considerably following roxadustat therapy (Figure 9).

#### 4.1.8 AEs

**FIGURE 10:**
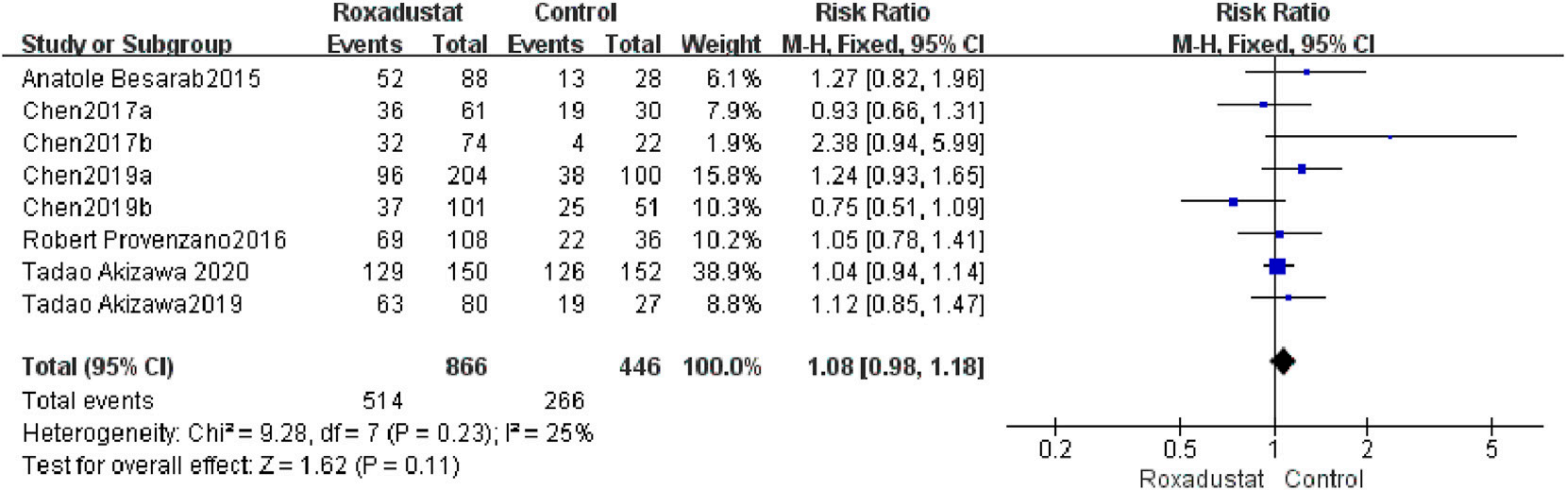
AEs.

A total of eight trials with a total of 1,312 patients compared the occurrence of AEs following treatment in the roxadustat group (*n* = 464) and the control group (*n* = 298), and the overall effect size [SMD = 1.08, 95%CI (0.98, 1.18), *p* = 0.0.11] was not statistically significant. The meta-analysis results showed that there was no difference in AEs following roxadustat treatment (Figure 10).

#### 4.1.9 SAEs

**FIGURE 11:**
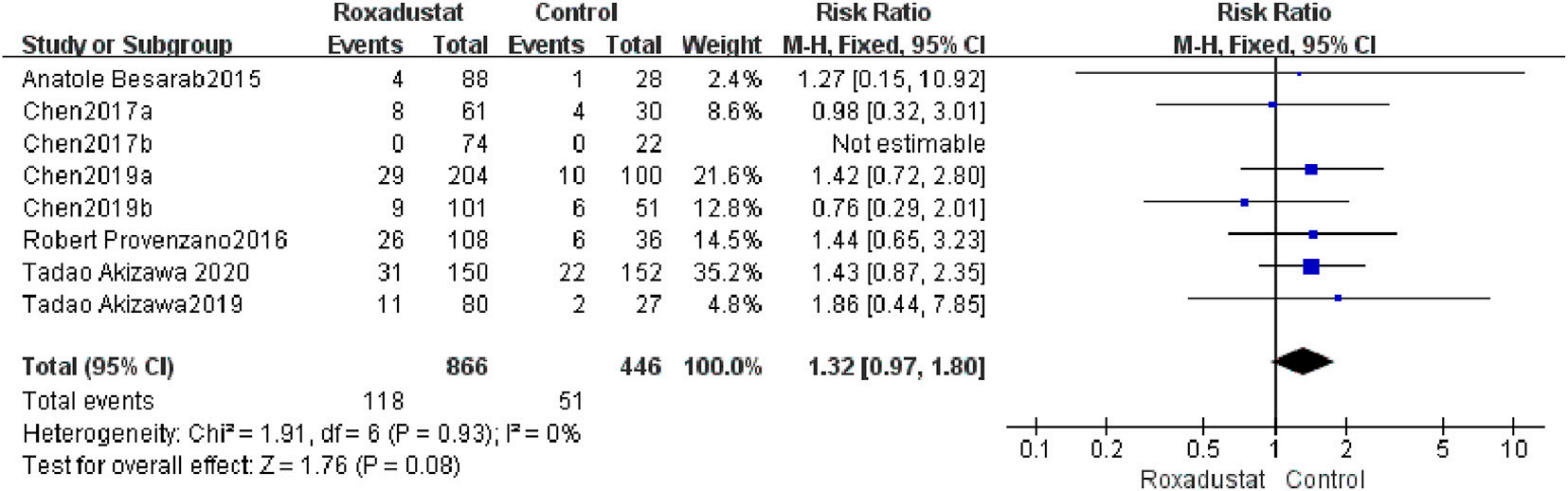
SAEs.

A total of eight trials with a total of 1,312 patients examined the changes in SAEs following treatment between the roxadustat group (*n* = 464) and the control group (*n* = 298), and the overall effect size [SMD = 1.32, 95%CI (0.97, 1.80), *p* = 0.08] was not statistically significant. The meta-analysis results revealed that there was no change in SAEs treated with roxadustat (Figure 11).

#### 4.1.10 Other Adverse Events

##### 4.1.10.1 Diarrhea

**FIGURE 12:**
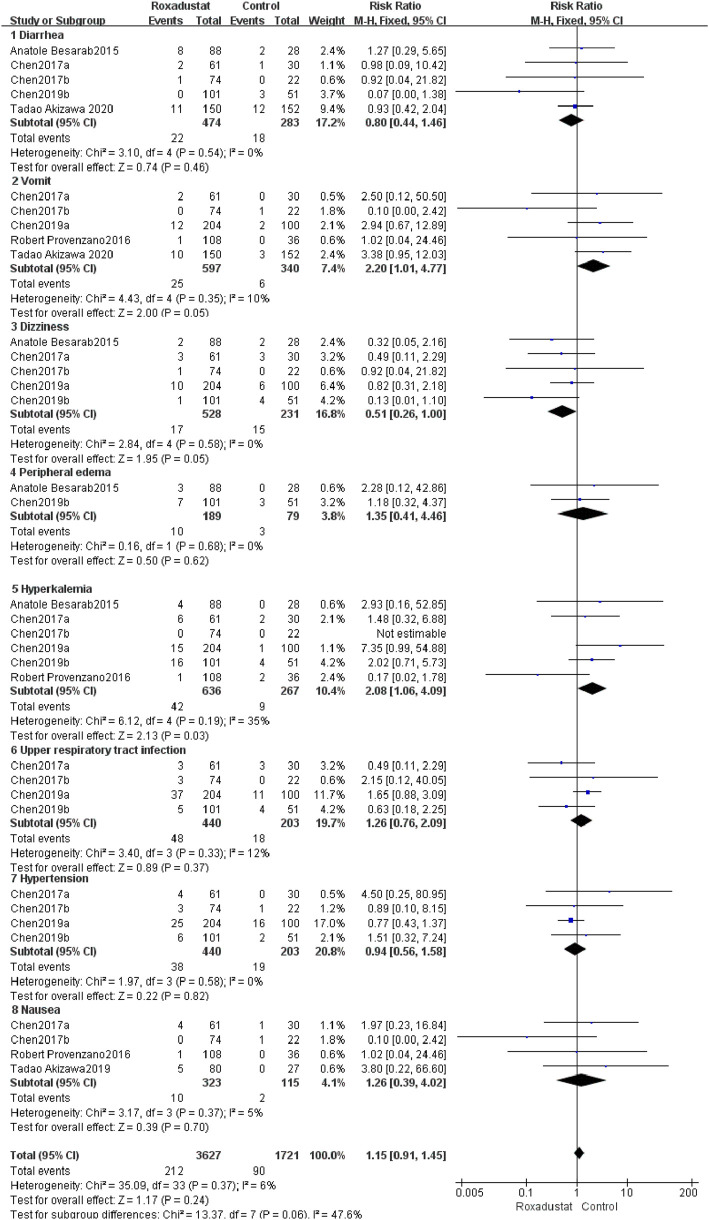
Other adverse events.

A total of five trials with 757 patients comparing diarrhea following therapy in the roxadustat group (*n* = 464) and the control group (*n* = 298), and the overall effect size [SMD = 0.8, 95%CI (0.44, 1.46), *p* = 0.46] did not show a statistically significant difference. According to the findings of a meta-analysis, roxadustat did not raise the risk of diarrhea in patients (Figure 12).

##### 4.1.10.2 Vomiting

A total of five trials with 937 patients compared the vomiting of the roxadustat group (*n* = 464) and the control group (*n* = 298) after therapy, and the combined effect size [SMD = 2.2, 95%CI (1.01, 4.77), *p* = 0.05] did not show a statistically significant difference. According to the findings of the meta-analysis, roxadustat did not increase the likelihood of vomiting in patients (Figure 12).

##### 4.1.10.3 Dizziness

A total of five trials with 759 patients compared the roxadustat group (n = 464) and the control group (*n* = 298) who reported dizziness after therapy, and the overall effect size [SMD = 0.51, 95%CI (0.26, 1.00), *p* = 0.05] did not show a statistically significant difference. According to the findings of a meta-analysis, roxadustat did not raise the risk of dizziness in patients (Figure 12).

##### 4.1.10.4 Peripheral edema

A total of two studies with a total of 268 patients examined the changes in SAEs following treatment in the roxadustat group (*n* = 464) and the control group (*n* = 298), and the combined effect size was not statistically significant [SMD = 1.35, 95%CI (0.41, 4.46), *p* = 0.62]. According to the findings of a meta-analysis, roxadustat did not raise the risk of peripheral edema in patients (Figure 12).

##### 4.1.10.5 Hyperkalemia

Six trials with a total of 903 patients examined the occurrence of hyperkalemia in the roxadustat group (*n* = 464) and the control group (*n* = 298) following therapy, and the overall effect size [SMD = 2.08, 95 %CI (1.06, 4.09), *p* = 0.03] indicated a statistically significant difference. According to the findings of a meta-analysis, roxadustat may raise the risk of hyperkalemia in patients (Figure 12).

##### 4.1.10.6 Upper respiratory tract infection

A total of four trials with 643 patients examined the occurrence of upper respiratory tract infections following therapy in the roxadustat group (*n* = 464) and the control group (*n* = 298), and the overall effect size [SMD = 1.26, 95% CI (0.76, 2.09, *p* = 0.37] was not statistically significant. According to the findings of a meta-analysis, roxadustat did not increase the likelihood of patients developing upper respiratory tract infection (Figure 12).

##### 4.1.10.7 Hypertension

A total of four trials with 643 patients examined the incidence of hypertension following therapy in the roxadustat group (*n* = 464) with the non-roxadustat control group (*n* = 298), and the cumulative effect size [SMD = 0.94, 95%CI (0.56, 1.58), *p* = 0.82] was not statistically significant. According to the findings of the meta-analysis, roxadustat did not enhance the risk of hypertension in patients (Figure 12).

##### 4.1.10.8 Nausea

A total of four trials with 438 patients comparing nausea following therapy in the roxadustat group (*n* = 464) and the control group (*n* = 298), and the cumulative effect size [SMD = 1.26, 95%CI (0.39, 4.02), *p* = 0.7] was not statistically significant. According to the findings of the meta-analysis, Roxadustat did not raise the risk of nausea in patients (Figure 12).

### 4.2 Subgroup Analysis

We conducted separate subgroup analyses for outcome indicators with significant heterogeneity by area and control group, although heterogeneity remains due to the small number of included studies (Tables 2, 3, 4).

**TABLE 2:** Subgroup analysis of study region.

Outcome	Region	DD/NDD	No.of trials	No.of patients	SM	RR (95%CI)	I2	*p*
HB	CHN	DD	2	243	REM	2.94 (2.35, 3.53)	86	<0.0001
	JP	DD	1	107	REM	1.70 (0.87, 2.53)	—	<0.00001
Transferrin	CHN	DD	2	336	REM	0.74 (−0.23, 1.72)	91	0.14
	JP	DD	1	301	REM	0.91 (0.67, 1.14)	—	<0.00001
	CHN	NDD	2	243	REM	1.62 (1.36, 1.97)	0	<0.00001
	JP	NDD	1	107	REM	0.74 (0.29, 1.19)	—	0.001
TIBC	CIN	DD	2	336	REM	1.10 (0.81, 1.4)	20	<0.00001
	USA	DD	2	125	REM	0.93 (−0.43, 2.28)	87	0.18
	JP	DD	1	301	REM	0.88 (0.65, 1.12)	—	<0.00001
	CHN	NDD	2	243	REM	1.68 (1.38, 1.99)	0	<0.00001
	USA	NDD	1	96	REM	1.17 (0.67, 1.67)	—	<0.00001
	JP	NDD	1	107	REM	0.79 (0.34, 1.24)	—	0.0006
Hepcidin	CHN	NDD	2	243	REM	2.54 (−3.83, 8.90)	99	0.43
	USA	NDD	2	87	REM	−1.28 (−1.75,−0.82)	0	<0.00001
	JP	NDD	1	107	REM	−0.4 (−0.83, 0.04)	—	0.64

HB, Hemoglobin; CHN, China; JP, Japan; USA, Unitied States; TIBC, total iron binding capacity; REM, Random-effects model; SM, statistical method.

**TABLE 3:** Subgroup analysis of control.

Outcome	Control	DD/NDD	No.of trials	No.of patients	SM	RR (95%CI)	I2	*p*
Transferrin	Epoetin alfa	DD	2	336	REM	0.74 (−0.23, 1.72)	91	0.14
	DA	DD	1	301	REM	0.91 (0.67, 1.14)	—	<0.00001
TIBC	Epoetin alfa	DD	4	461	REM	1.02 (0.52, 1.52)	76	<0.0001
	DA	DD	1	301	REM	0.88 (0.65, 1.12)	—	<0.00001

DA, darbepoetin alfa; REM, random-effects model; SM, statistical method.

**TABLE 4:** publication bias.

Outcome	No. of trials	Begg’s test	Egeer’s test	Publication bias
HB	5	*p* = 0.462	*p* = 0.136	No
Iron	8	*p* = 0.902	*p* = 0.608	No
TSAT	9	*p* = 0.917	*p* = 0.459	No
Ferritin	9	*p* = 0.602	*p* = 0.078	No
TIBC	9	*p* = 0.251	*p* = 0.349	No
Transferrin	6	*p* = 0.707	*p* = 0.185	No
Hepcidin	10	*p* = 0.007	*p* = 0.024	Yes
AEs	8	*p* = 0.452	*p* = 0.027	Yes
SAEs	8	*p* = 0.452	*p* = 0.422	Yes

### 4.3 Publication Bias

Begg’s funnel plot and Egger’s regression were used to examine publication bias, and our research indicated no publication bias except for hepcidinAEs and SAEs, where there may have been publication bias.

## 5 Discussion

This analysis comprised 8 RCTs (7 articles) published between 2015 and 2020, with a total of 1,364 individuals aged 18–80 years with chronic kidney disease anemia. According to the study’s features, the dosing range for roxadustat was 0.7 mg/kg to 2.3 mg/kg three times a week; the measurement of Hb, transferrin, hepcidin, ferritin, TSAT, TIBC, and serum iron before and after the observed changes; and adverse events. The study’s findings demonstrated that treatment with roxadustat resulted in considerably larger average increases in hemoglobin, TIBC, and serum iron in both the dialysis and nondialysis groups than in the control group, a finding that was similar with the findings of Qie et al. (2021) and others. When we included the Akizawa et al. (2020b) study, we discovered that roxadustat treatment resulted in significantly higher average increases in transferrin in both the dialysis and nondialysis groups than in the control group; roxadustat could reduce ferritin, hepcidin, and TSAT in the nondialysis group, but not in the dialysis group. The change in serum iron was substantially greater in the dialysis group than in the control group, while the difference was not significant in the nondialysis group. There was no significant difference in AEs and SAEs between the roxadustat group and the control group in terms of safety. Roxadustat raises the likelihood of hyperkalemia in patients, according to other adverse effects.

Renal anemia is mostly caused by a relative or absolute deficit of EPO and iron, which is produced by a loss in renal function and a disruption in iron metabolism (Xu et al., 2011)^.^ Furthermore, uremic toxins, inflammation and infection, and starvation might worsen renal anemia (Verdalles et al., 2011). More than 30%–50% of patients present serological evidence of an active inflammatory state, such as CRP, interleukin-1, interleukin-6, and tumor necrosis factor. (Owen and Lowrie, 1998; Stenvinkel et al., 2002; Nakanishi et al., 2019)^.^ Numerous studies have demonstrated that inflammation influences the development of renal anemia, inhibits the production of endogenous EPO and iron absorption and utilization, raises hepcidin levels, causes iron metabolism problems, and accelerates the clearance of red blood cells by macrophages. Microinflammation and iron overload are frequent in CKD patients and can cause serum hepcidin expression to increase. The risk of infection and inflammation is enhanced in DD patients, and the amount of hepcidin is much greater than in healthy persons (Kuragano et al., 2010). Excess hepcidin can block iron absorption in the gut as well as iron release and output by the liver and reticulocytes, resulting in iron metabolism problems, decreased Hb production, and worsening anemia in CKD patients (Kali et al., 2015). Current treatment options for renal anemia mostly involve iron supplements and erythropoietin replacement therapy (Jjv et al., 2012).

Roxadustat is an oral HIF stabilizer and a hypoxia inducible factor prolyl hydroxylase inhibitor. Roxadustat, a hypoxia inducible factor prolyl hydroxylase inhibitor (HIF-PHI), has been shown in the literature to heal anemia and improve iron metabolism (Haase, 2013; Maxwell and Eckardt, 2016). The transcription factor hypoxia inducible factor (HIF) Hypoxia can activate the transcription of the EP0 gene, upregulate transferrin and receptors, raise transferrin levels, and so on. Iron consumption boosts hemoglobin synthesis, decreases hepcidin levels, and organizes and enhances red blood cell formation of the two main ingredients of EPO and iron, so alleviating anemia (Kuragano et al., 2010; Verdalles et al., 2011; Xu et al., 2011; Dhillon, 2019). The primary enzyme that inactivates HIF is prolyl hydroxylase. Anemia can be repaired by blocking prolyl hydroxylase and lowering HIF inactivation to maintain high levels of this transcription factor.

Clinical trial investigations have demonstrated that roxadustat (Besarab et al., 2015b) reduces hepcidin in NDD-CKD patients, which is compatible with the results stated in this article. After 4 weeks of dialysis, the amount of hepcidin in individuals with CKD fell significantly (Chen et al., 2019a). In a meta-analysis, we discovered that roxadustat reduced hepcidin levels in patients with renal anemia. Lowering hepcidin levels is critical for proper erythropoiesis. Hepcidin is an essential measure of iron bioavailability and can boost erythropoietin to within the physiological range since it plays a vital function in the delivery and use of iron. These data corroborate the conclusions of our meta-analysis.

Finally, roxadustat is more successful and safer in the treatment of anemia in CKD patients. The overall quality of the studies included in this research was quite excellent, and the majority of the combined indicators were heterogeneous. After removing evident clinical heterogeneity, the results were steady and trustworthy, according to the random effects model and the fixed effects model analysis. The number of patients involved, however, was restricted, and only 1 year of safety data was provided. At the same time, fewer studies are presently included; for example, there are just two papers in the study of hemoglobin alterations. A large-scale worldwide multicenter phase IV trial is still needed to prove the safety of long-term therapy. As a result, more high-quality, standardized research is required to further evaluate the safety and clinical efficacy of roxadustat and offer a credible foundation for developing scientific and appropriate treatment methods for enhancing efficacy and minimizing hazards.

## Data Availability

The original contributions presented in the study are included in the article/Supplementary Material, further inquiries can be directed to the corresponding author.
